# Disrupting the TGF-β-regulated epithelial-mesenchymal transition, apoptotic and autophagic phenotypes of 3D glioblastoma spheroids via glycolytic inhibition

**DOI:** 10.37349/etat.2026.1002364

**Published:** 2026-03-30

**Authors:** Maellis Payet-Desruisseaux, Alain Zgheib, Bogdan Alexandru Danalache, Michel Desjarlais, Borhane Annabi

**Affiliations:** University of Saskatchewan, Canada; ^1^Laboratoire d’Oncologie Moléculaire, Département de Chimie, Université du Québec à Montréal, Montreal, QC H3C 3P8, Canada; ^2^HMR-RC, Université de Montréal, Montreal, QC H3T 1E2, Canada

**Keywords:** glioblastoma, TGF-β, epithelial-to-mesenchymal transition, 2-deoxy-*D*-glucose, glycolytic inhibition, apoptosis, autophagy

## Abstract

**Aim:**

Glioblastoma (GBM), a rare, highly aggressive and chemoresistant brain cancer, exhibits profound metabolic plasticity that relies, in part, on aberrant transforming growth factor-β (TGF-β) signaling. Such plasticity was recently associated with TGF-β-regulated apoptosis and autophagy. Here, we questioned whether TGF-β-regulated apoptotic/autophagic phenotypes are recapitulated in a preclinical in vitro 3D spheroid culture model of human U87 GBM-derived cells, and how metabolic alterations affect such phenotypes.

**Methods:**

3D U87 spheroids were cultured using the hanging drop method. Western blotting was used to assess protein expression, while RT-qPCR was used to assess gene expression levels.

**Results:**

3D spheroids exhibited decreased AKT phosphorylation, and increased TGF-β, fibronectin, and Smad2 phosphorylation, indicative of both cell death signaling and epithelial-mesenchymal transition molecular signatures. 2-Deoxy-*D*-glucose (2DG), a glycolytic inhibitor, depleted ATP dose-dependently (30–300 μM) and prevented those increases both at the protein and transcriptional levels. This was also observed in 3D spheroids upon TGF-β transient siRNA-mediated silencing or when TGF-βR1 kinase activity was inhibited by galunisertib. Transcriptomic profiling revealed shared upregulation of apoptosis-related (*BCL2*, *CASP7*, *FAS*, *FASLG*, *GADD45A*) and autophagy-related (*ATG7*, *ATG16L1*, *IRGM*, *PIK3C3*, *ULK1*) genes in response to TGF-β or upon 3D spheroid formation. 2DG, transient silencing of TGF-β, or galunisertib treatment prevented these increases.

**Conclusions:**

3D spheroids require ATP and a TGF-β/TGF-βR1 autocrine signaling axis to recapitulate the apoptosis/autophagy phenotypes. Combining glycolysis inhibition with TGF-β signaling inhibition could offer a promising therapeutic strategy for this rare and lethal brain cancer.

## Introduction

Glioblastoma (GBM) remains one of the most aggressive and treatment-resistant brain tumors, with a poor prognosis despite multimodal therapeutic approaches [1, 2]. A hallmark of GBM is its reliance on metabolic reprogramming, including enhanced glycolysis, to fuel rapid proliferation and survival under hypoxic conditions [3, 4]. Other important molecular alterations, notably in survival signaling, have been reported to actively contribute to GBM progression, in part driven by aberrant transforming growth factor-β (TGF-β) signaling, an important multifaceted pathway that acts as a dual pro-angiogenic and cell death regulator [5, 6]. Indeed, while TGF-β signaling can enhance invasiveness, immune suppression, and the maintenance of stem-like phenotypes [7], it can also trigger cell death apoptosis/autophagy processes to ensure survival under stress and contribute to therapy resistance [8].

The contribution of TGF-β has been inferred to play a crucial role in triggering the epithelial-mesenchymal transition (EMT) process. This cellular program regulates epithelial cellsʼ loss of polarity and intercellular connections, allowing cells to adopt a mesenchymal phenotype reflecting increased invasiveness. When TGF-β binds its receptor complex made up of TGF-βR1 and TGF-βR2, phosphorylation of receptor-regulated Smads (Smad2/3) is triggered and regulates the transcription of genes related to EMT [9–11]. Additionally, TGF-β activates several non-canonical signaling pathways, including MAPK (ERK, JNK, p38), PI3K/AKT, and Rho GTPases, which work together to remodel the cytoskeleton, stabilize transcription factors that induce EMT (like Snail, Slug, ZEB1/2, Twist), and boost cell migration [12]. Interestingly, TGF-βʼs role in tumor development appears context-dependent, as it can act as a tumor suppressor in the early stages by promoting apoptosis [8]. Therefore, TGF-β serves as a key molecular switch that orchestrates epithelial plasticity and malignant transformation through the coordinated activation of transcriptional and signaling networks.

Studies have shown that 2-deoxy-*D*-glucose (2DG), a glucose analog that inhibits glycolysis and depletes ATP levels [13], impairs energy-dependent signaling cascades such as TGF-β [14]. This leads to attenuated TGF-β-driven transcriptional programs and disruption of tumor-promoting phenotypes [15]. By investigating this synergistic interplay, the current study aims to establish mechanistic evidence of how metabolic disruption through 2DG could alter the response to TGF-β signaling in GBM cells, potentially laying the groundwork for novel combinatorial treatments in high-grade gliomas. By combining metabolic inhibition with TGF-β suppression, novel approaches could synergistically impair GBM cell survival and malignancy [16, 17]. Therefore, the rationale behind this strategy is to exploit the interdependence between energy metabolism and oncogenic signaling to achieve deeper therapeutic responses [18]. The study further aims at providing new mechanistic insights and evaluating the pharmacological relevance of targeting these convergent pathways in preclinically validated in vitro 3D spheroid models.

Recent evidence suggests that this metabolic/autophagic/apoptotic axis is tightly regulated, as GBM stem-like cells were found to upregulate autophagy in response to glycolytic inhibition, thereby maintaining stemness and resisting apoptosis [19]. Moreover, studies have shown a complex bidirectional feedback loop, where inhibition of autophagy enhances glycolytic activity, and vice versa, underscoring the functional interplay between glucose-6-phosphate (G6P)-driven metabolic stress and autophagic activation [20]. Here, we further questioned whether 1) this connection can be recapitulated in 3D spheroid cultures of GBM-derived cells, which show a stem cell phenotype [21, 22], and 2) attempted to provide a deeper mechanistic insight into how GBM cells can adapt to metabolic perturbations that would allow them to resist treatment.

## Materials and methods

### Reagents

Sodium dodecylsulfate (SDS), bovine serum albumin, dimethylsulfoxide (DMSO), TGF, and galunisertib were sourced from Sigma (Oakville, ON, Canada). Electrophoresis reagents came from Bio-Rad (Mississauga, ON, Canada). The enhanced chemiluminescence reagent was from Amersham Pharmacia Biotech (Baie d’Urfé, QC, Canada). Micro bicinchoninic acid protein assay reagents were from Pierce (Rockford, IL, USA). The rabbit polyclonal antibody against GRP78 (SC-13968) and fibronectin (SC-8422) was from Santa Cruz Biotechnology (Dallas, TX, USA). Polyclonal antibodies against TGF-β (56E4, 3709 S), AKT (11521, 9272 S), phospho-AKT Ser473 (12604, 4060 S), Snail (C15D3, 3879 S), Smad2 (D43B4, 5339 S), Smad3 (C67H9, 9523 S), phospho-Smad2 Ser465/467 (138D4, 3108 S), and phospho-Smad3 Ser423/425 (C25A9, 9520 S) were from Cell Signaling Technology Inc. (Danvers, MA, USA). The monoclonal antibody against GAPDH (D4C6R, 97166 S) was from EMD Millipore (Oakville, ON, Canada). Horseradish peroxidase-conjugated secondary antibodies were from Jackson ImmunoResearch Laboratories (West Grove, PA, USA).

### Cell culture

Human U87 GBM-derived cells and culture media were from the American Type Culture Collection (ATCC; Manassas, VA, USA). According to the manufacturer, mycoplasma contamination was eliminated in September 1975 from these cells. This was confirmed bi-annually in our laboratory, and we found no contamination. The STR profiling, Y-chromosome paint, and Q-band assay, provided by the manufacturer, confirm that the cell line was male in origin, and likely a GBM of CNS origin (https://www.atcc.org/products/htb-14). Cells were cultured as 2D monolayers without antibiotics in Dulbecco’s modified Eagle’s medium (DMEM) containing 10% fetal bovine serum (FBS). Cells were kept subconfluent and used over a maximum of eight passages, with a 1:5 bi-weekly split, in a humidified incubator at 37°C with 5% CO_2_.

### Purine nucleotide measurements

Intracellular 2DG-mediated ATP depletion was measured as described elsewhere using HPLC separation of purine nucleotides carried out with an Agilent 1260 Infinity system (Agilent Technologies) with the multiple-wavelength detector set to measure absorbance at 248, 254, and 262 nm simultaneously. Quantification of nucleotides was performed by peak integration of the area under the curve and verified using external standards of known concentration as determined by the molar extinction coefficient [23].

### Formation of 3D hanging drop spheroids

The formation of 3D spheroids was obtained using the hanging drop technique, which involves suspended inverted cell droplets and culturing cells in 100 mm non-adherent Petri dishes (low-attachment plates) to prevent substrate adhesion as previously described [21, 24]. The lids were removed, and 10 mL of sterile water was added to the bottom of each dish to create a hydration chamber. Using a multichannel pipette, 38 μL drops of cell suspension (at densities of 38,000 cells per drop) were carefully dispensed onto the inner surface of the inverted lid, ensuring sufficient spacing to prevent contact between drops. Up to 40 drops were placed per lid. The lid was then inverted over the water-filled bottom chamber and incubated at 37°C in a humidified atmosphere with 5% CO_2_ and 95% humidity for 5 days. The droplets were monitored daily under a microscope to assess cellular aggregate formation. Spheroids were either analyzed directly or transferred to 96-well round-bottom plates (FALCON #351177) coated with poly(2-hydroxyethyl methacrylate) [poly-HEMA] (P3932, Sigma-Aldrich, Oakville, ON, Canada) to prevent cell adhesion to the growth surface. Each well contained 250 μL of fresh complete medium.

### Microscopy and analysis of spheroids

Spheroid morphology was examined under a Nikon Eclipse TE2000-U inverted microscope (Nikon Instruments Inc., Melville, NY, USA). Phase-contrast micrographs were taken with a Q Imaging QICAM-IR Fast 1394 Digital CCD camera using the NIH μManager software (National Institutes of Health, Bethesda, MD). For 96-well plates, an Agilent BioTek Cytation 5 cell imaging multimode reader with a 4× objective was used to image the entire well using the bright field imaging channel using Gen5 software. Quantification of spheroid size was performed using QuPath 0.5.1 along with the Segment Anything Model (SAM) extension, a validated tool that employs deep learning techniques to segment objects within images by identifying their contours and features [25].

### Western blot

Origin of electrophoresis reagents, total cell lysis procedure, SDS-polyacrylamide gel electrophoresis, electrotransfer to low-fluorescence polyvinylidene difluoride membranes, and immunodetection were conducted as described previously in detail [26]. Immunoreactive material was visualized by enhanced chemiluminescence.

### Transient transfection and RNA interference

U87 cells were transiently transfected with small interfering RNA (siRNA) sequences using Lipofectamine 2000 transfection reagent (Thermo Fisher Scientific). Gene silencing was performed using 20 nM siRNA against TGF-β (Hs_TGFB1_2 FlexiTube siRNA GeneGlobe ID: SI00013601), or scrambled sequences (AllStar Negative Control siRNA, 1027281) synthesized by QIAGEN (Valencia, CA, USA) and annealed to form duplexes. Gene silencing efficacy was assessed by real-time quantitative PCR (RT-qPCR) as described below.

### Total RNA extraction, cDNA synthesis, and RT-qPCR

Total RNA was extracted from 2D cell monolayers and 3D spheroids using TRIzol, following the manufacturer’s recommendations (Life Technologies, Gaithersburg, MD, USA). Total RNA concentration was measured using a NanoPhotometer P330 (Implen), and 2 μg was reverse-transcribed into cDNA using a high-capacity cDNA reverse transcription kit (Applied Biosystems; Foster City, CA, USA). Samples were prepared using primer sets and SsoFast EvaGreen Supermix (Bio-Rad; 1725204). The following QuantiTect primer sets were provided by QIAGEN: FN1 (Hs_FN1_1_SG QT00038024), SNAI1 (Hs_SNAI1_1_SG QT00010010), TGF-β (Hs_TGFB1_1_SG QT00000728), VEGF (Hs_VEGFA_1_SG QT01010184), GLUT1 (Hs_SLC2A1_1_SG QT00068957), HIF-1α (Hs_HIF1A_1_SG QT00083664), GAPDH (Hs_GAPDH_2_SG QT01192646) and PPIA (Hs_PPIA_4_SG QT01866137). Gene expression was quantified by RT-qPCR on a CFX Connect (Bio-Rad) with the Bio-Rad CFX Manager software version 3.0. The relative RNA quantities of each target gene were normalized against two housekeeping genes, *GAPDH* and *PPIA*, using the standard 2^–ΔΔCq^ method.

### Human cell death pathway and EMT cell profiler PCR arrays

Premade RT^2^ Profiler PCR arrays for Human Cell Death (PAHS-212Z) and for Human Epithelial-Mesenchymal Transition (PAHS-090Z) were purchased from QIAGEN and were used following the manufacturer’s instructions. Briefly, the genomic DNA was eliminated before reverse-transcribing 4 μg of total RNA using the RT^2^ First Strand Kit (QIAGEN, 330404). Each plate was used to assess one cDNA sample prepared with the RT^2^ SYBR Green qPCR Master Mix (QIAGEN, 330502). The relative expression analysis of 84 genes, as well as controls, was done through the GeneGlobe Analysis Center, a website provided by QIAGEN (https://geneglobe.qiagen.com/us/analyze), using the standard fold change 2^–ΔΔCq^ method. Fold regulation was used in the figures; for upregulated genes, fold regulation is the same as fold change, but for downregulated genes, fold regulation is calculated as 1/(fold change).

### Protein-to-protein interactions

The STRING database v11 (https://www.string-db.org/) was used to identify and build protein-to-protein interaction networks [27], with a confidence score of 0.4. The maximum number of interactions shown was set to 10 to help with the readability of the predictions.

### Statistics

All statistical analyses were conducted using the GraphPad Prism 7 software (Dotmatics). Data and error bars are expressed as the mean ± standard error of the mean from three independent experiments, unless otherwise specified. Hypothesis testing was performed using the Kruskal‑Wallis test followed by a Dunn’s post-hoc test (> 2 groups). *P* < 0.05 (*) values are considered significant and are indicated in the figures.

## Results

### Glycolytic inhibition triggers metabolic stress and impairs EMT, TGF-β signaling, and hypoxia-related gene expression in 3D spheroids

**Figure 1:**
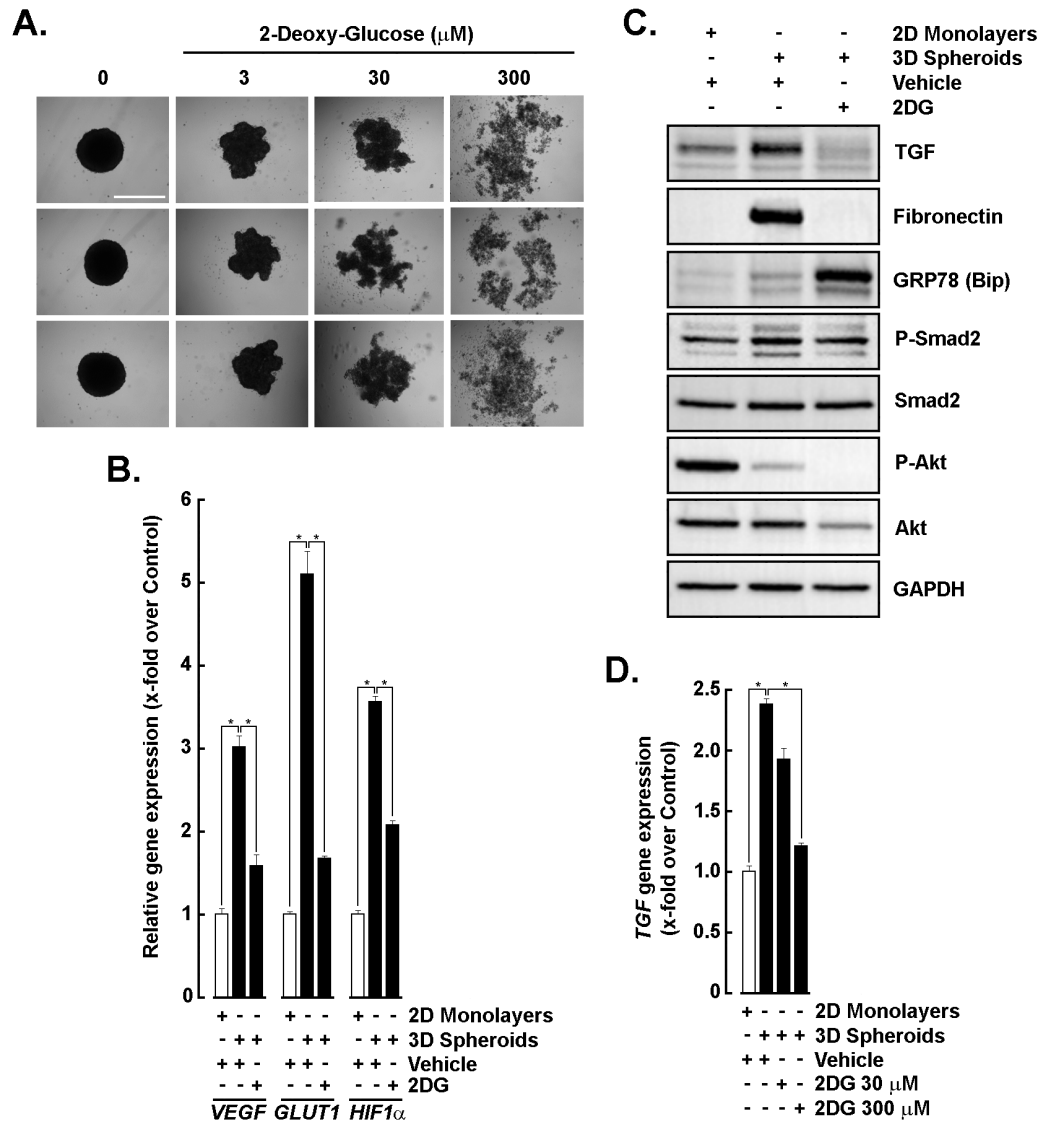
**Glycolytic inhibition alters EMT and triggers stress signaling in 3D spheroids.** (**A**) Representative images of 3D spheroids treated with increasing concentrations of 2-deoxy-*D*-glucose (2DG; 0–300 μM). Scale bar = 1,000 μm. (**B**) Relative angiogenic gene expression of *VEGF*, *GLUT1*, and *HIF-1α* in 2D monolayers (white bars) and 3D spheroids (black bars) following vehicle (H_2_O) or 2DG (300 μM) treatment. (**C**) Western blot analysis of EMT markers (TGF-β, fibronectin), ER stress marker (GRP78/Bip), and signaling proteins (phospho-Smad2, Smad2, phospho-Akt, Akt) in 2D monolayers and 3D spheroids treated with vehicle or 2DG (300 μM). GAPDH served as a loading control. (**D**) RT-qPCR analysis of TGF-β mRNA expression in 2D monolayers and 3D spheroids under vehicle or 2DG treatment (30 and 300 μM). All RT-qPCR data are expressed as fold change relative to control and performed in triplicate from two independent experiments. *P* < 0.05 (*) values are considered significant. EMT: epithelial-mesenchymal transition; TGF-β: transforming growth factor-β; ER: endoplasmic reticulum.

ATP serves as the primary energy currency supporting cytoskeletal remodeling, cell–cell adhesion, and vesicular trafficking necessary for 3D spheroid compaction [22]. Therefore, investigating ATP requirements during the 3D in vitro GBM spheroid formation provides mechanistic insight into how energy metabolism may govern 3D tumor organization and acquisition of an invasive phenotype. Here, we assessed the impact of glycolytic inhibition on EMT in 3D spheroids treated with increasing concentrations of 2DG. Morphological analysis revealed that 2DG led to dose-dependent disruption of spheroid integrity, indicating reduced cell–cell adhesion under metabolic stress (Figure 1A). RT-qPCR confirmed higher *VEGF*, *GLUT1*, and *HIF-1α* transcript levels upon spheroid formation, indicating activation of hypoxia and angiogenesis adaptive responses (Figure 1B). Importantly, 2DG treatment was found to prevent these increases, supporting the spheroid-disrupting events as a consequence of ATP depletion (Figure 1B). Western blotting showed elevated TGF-β, fibronectin, and phospho-Smad2 (P-Smad2) expression in 3D spheroids compared to 2D monolayers, consistent with enhanced EMT (Figure 1C), and this was confirmed by increased other EMT biomarkers using gene array screens, including TGF-β signaling intermediates, ECM organisation proteins, and cell surface integrins (Table S1). While GRP78 expression increased upon spheroid formation, 2DG treatment further potentiated that effect and led to a decreased EMT phenotype, suggesting 2DG-mediated glycolytic inhibition altered TGF-β signaling pathways. Finally, along with its increased expression at the protein level in spheroids, *TGF-β* transcript levels were also reduced upon 2DG treatment (Figure 1D). These findings demonstrate that 3D culture promotes EMT and that glycolytic inhibition potentially alters TGF-β autocrine signaling. In addition, reduced AKT phosphorylation also appears indicative of a cell death phenotype (Figure 1C). Decreased total Akt and P-Akt in 3D spheroids can rationally be a reflection of biologically meaningful shifts driven by the 3D microenvironment. Spheroid architecture alters growth factor access, cell–matrix signaling, metabolic state, phosphatase activity, and subpopulation composition; any of these can reduce Akt protein abundance or phosphorylation while stemness phenotypes are maintained or regulated through alternative pathways [28]. Importantly, this suggests that glioma stemness in our model can be maintained through Akt‑independent or context‑dependent pathways.

### Disrupting the TGF-β/TGF-βR1 signaling axis impairs EMT and spheroid integrity

**Figure 2:**
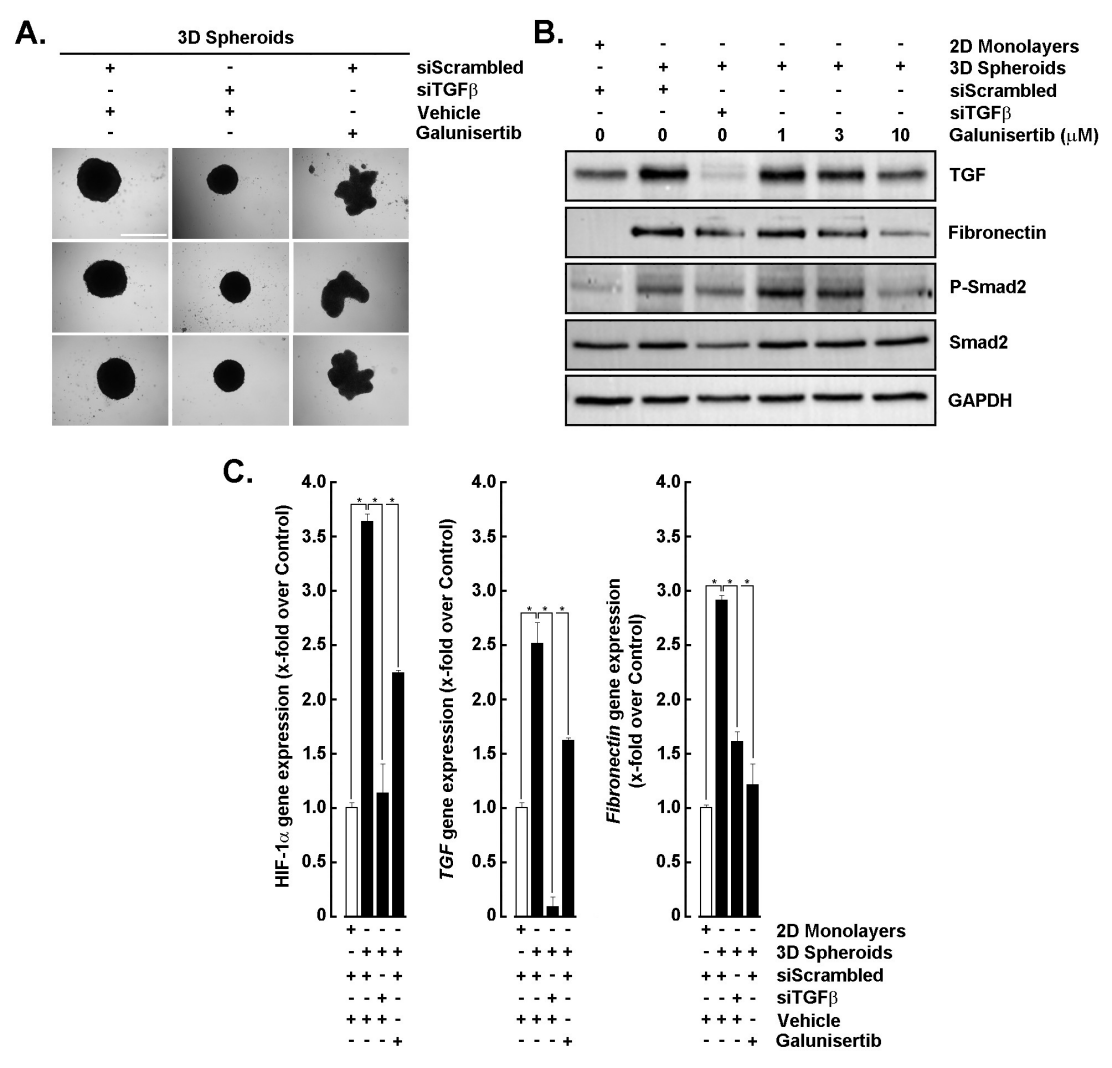
**Inhibition of TGF-β signaling reduces EMT marker expression in 3D spheroids.** (**A**) Representative images of 3D spheroids transfected with scrambled siRNA (siScrambled) or TGF-β siRNA (siTGFβ), then treated with vehicle (DMSO) or galunisertib (10 μM). Scale bar = 1,000 μm. (**B**) Western blot analysis of TGF-β, fibronectin, phospho-Smad2, and total Smad2 in 2D monolayers and 3D spheroids following siScrambled, siTGFβ, or galunisertib treatment (1–10 μM). GAPDH served as a loading control. (**C**) RT-qPCR analysis of *HIF-1α*, *TGF-β,* and *fibronectin* mRNA expression in 2D monolayers and 3D spheroids under the indicated conditions. All RT-qPCR data are expressed as fold change relative to control and performed in triplicate from two independent experiments. *P* < 0.05 (*) values are considered significant. TGF-β: transforming growth factor-β; EMT: epithelial-mesenchymal transition.

To determine the role of TGF-β/TGF-βR1 signaling in EMT within 3D spheroid cultures, cells were subjected to siRNA-mediated TGF-β knockdown (siTGFβ) or treated with galunisertib, a TGF-βR1 inhibitor [29, 30]. Morphological analysis showed that control spheroids (siScrambled) remained compact, whereas galunisertib treatment disrupted spheroid integrity and siTGFβ also reduced spheroid size (Figure 2A). Western blotting revealed elevated TGF-β and fibronectin expression in 3D spheroids compared to 2D monolayers, which were markedly reduced by siTGFβ and dose-dependent galunisertib treatment (Figure 2B). Both strategies targeting TGF-β downstream activity further led to a strong decrease in P-Smad2 levels, confirming effective inhibition of a potential TGF-β/TGF-βR1 signaling axis (Figure 2B). Consistent with these findings, RT-qPCR analysis demonstrated significant downregulation of *TGF-β* and *fibronectin* transcripts following siTGFβ and galunisertib exposure (Figure 2C, middle and right panels). Interestingly, spheroid-induced expression of *HIF-1α* was also altered (Figure 2C, left panel), as well as *VEGF* and *GLUT1* in accordance with spheroid disruption (not shown). These results suggest that TGF-β signaling is critical for EMT induction and maintenance in 3D spheroids, and its inhibition attenuates EMT-associated markers and signaling activity.

### Kinetics of canonical TGF-β signaling in GBM monolayers

**Figure 3:**
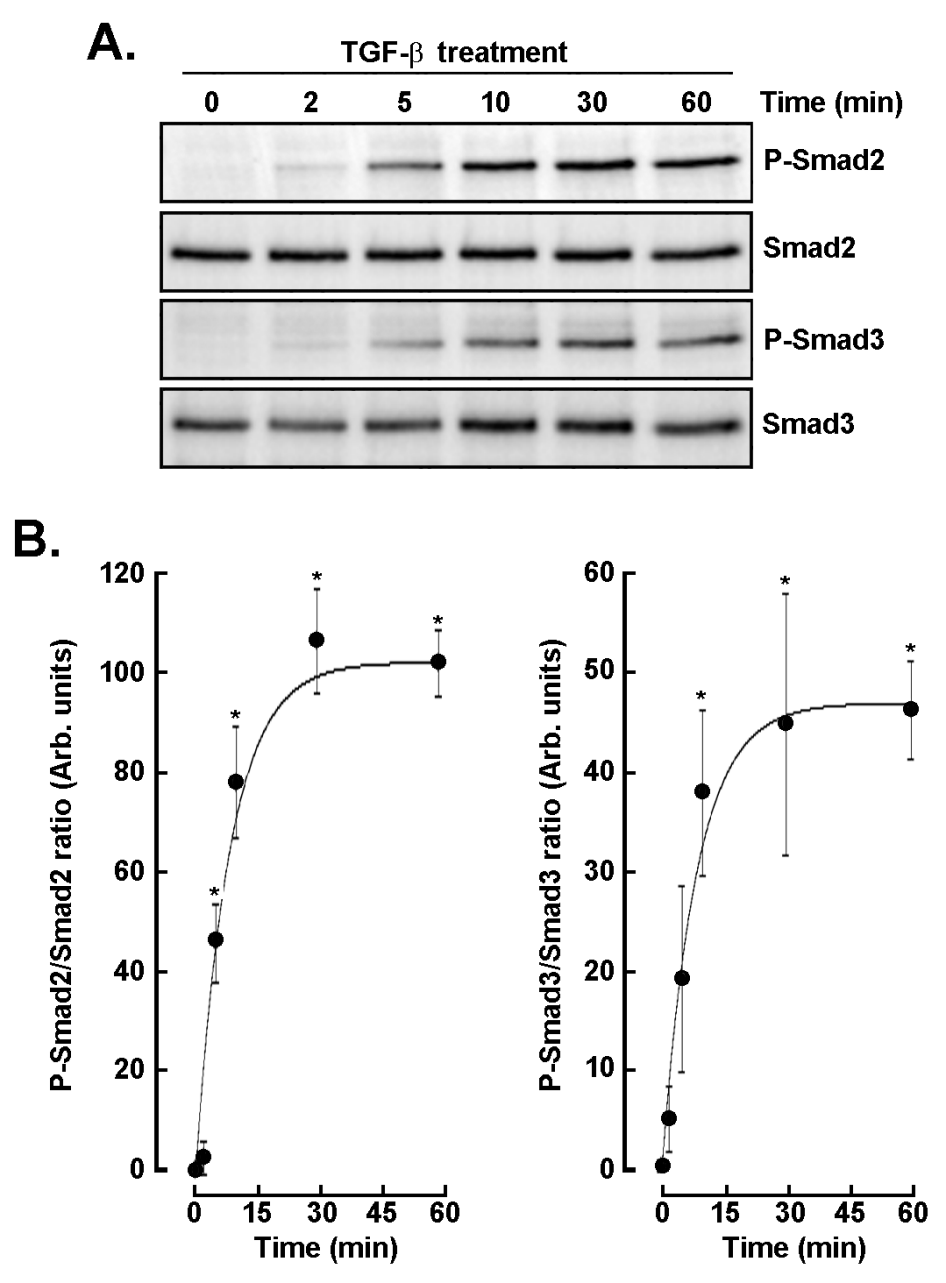
**Time-dependent phosphorylation of Smad2 and Smad3 following TGF-β stimulation.** (**A**) Western blot analysis of phospho-Smad2 (P-Smad2), total Smad2, phospho-Smad3 (P-Smad3), and total Smad3 in cells treated with TGF-β (30 ng/mL) for the indicated time. (**B**) Quantitative densitometric analysis of P-Smad2/Smad2 and P-Smad3/Smad3 ratios over time. Data are expressed as arbitrary units (mean ± SD) and are representative of three independent experiments. *P* < 0.05 (*) values are considered significant. TGF-β: transforming growth factor-β.

Given that autocrine TGF-β-mediated signaling was already involved in 3D spheroids, we decided to characterize the dynamics of TGF-β signaling through phosphorylation of Smad2 and Smad3 over time, in 2D GBM cell monolayers. Western blot analysis revealed rapid phosphorylation of both Smad2 and Smad3 within 2–5 min (Figure 3A), reaching near-maximal levels by 30 min and remaining sustained for up to 60 min, while total Smad2 and Smad3 levels remained unchanged. Quantitative analysis confirmed a sharp increase in P-Smad2/Smad2 and P-Smad3/Smad3 ratios during these first 30 min, then followed by a plateau, indicating rapid and stable activation of canonical TGF-β signaling (Figure 3B). These results suggest that Smad activation, a key mediator of cellular migration, proliferation, and differentiation processes, is also required for 3D spheroid regulation during an autocrine TGF-β process.

### Glycolytic inhibition impairs TGF-β-driven Smad2/3 activation and chemotaxis

**Figure 4:**
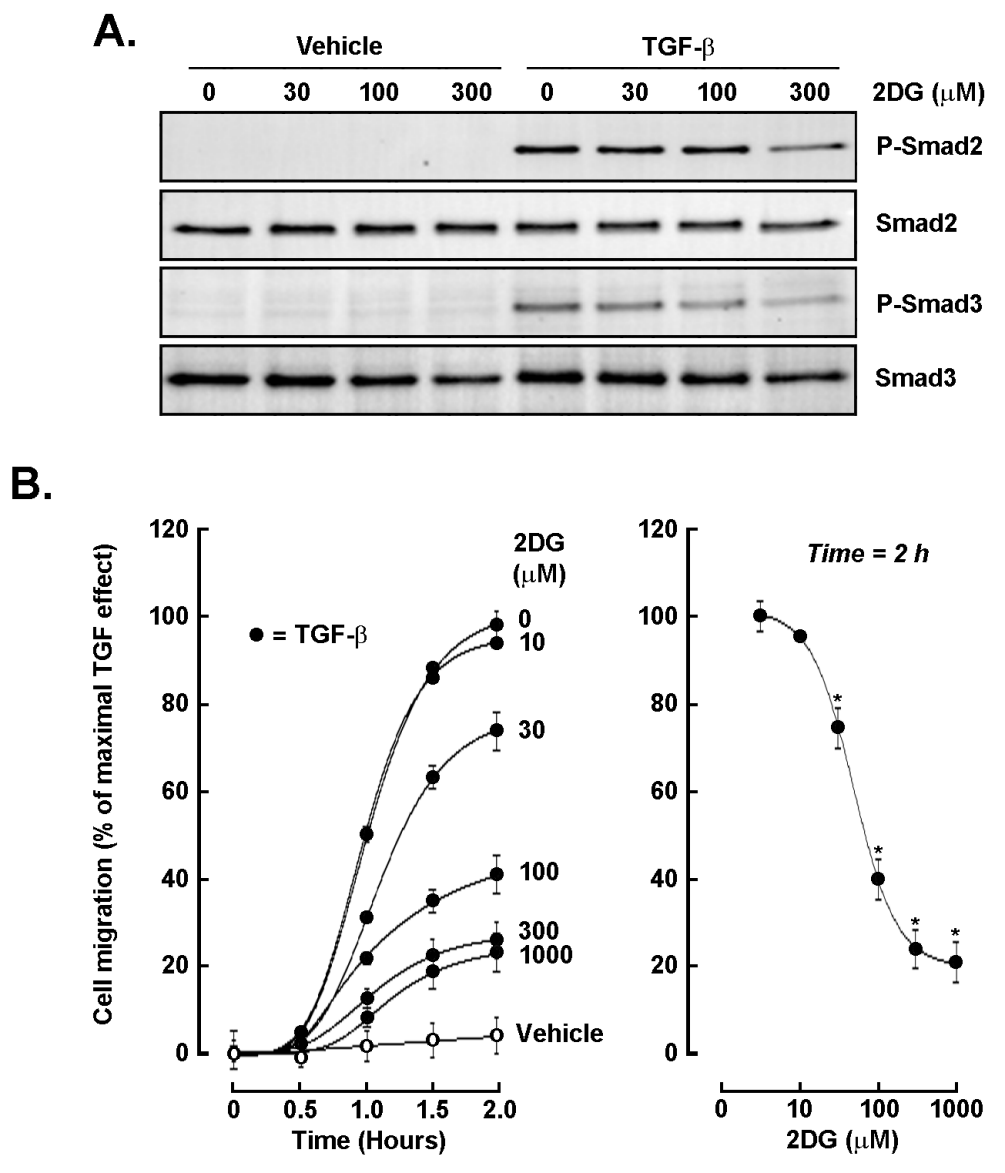
**Glycolytic inhibition attenuates TGF-β-induced Smad phosphorylation and cell migration.** (**A**) Western blot analysis of phospho-Smad2 (P-Smad2), total Smad2, phospho-Smad3 (P-Smad3), and total Smad3 in cells treated with vehicle or TGF-β (30 ng/mL) in the presence of increasing concentrations of 2-deoxy-*D*-glucose (2DG; 0–300 μM). (**B**) Quantitative cell migration assay showing TGF-β-induced migration over 2 h (closed circles) vs. unstimulated cells (open circles), and its dose-dependent inhibition by 2DG (left panel). The right panel summarizes migration at the 2-h time point, demonstrating near-complete suppression at ≥ 300 μM 2DG. Assay was performed in triplicate, and data are representative of two independent experiments. Data are expressed as a percentage of the TGF-β-stimulated control (mean ± SD). *P* < 0.05 (*) values are considered significant. TGF-β: transforming growth factor-β.

In order to address the potential role for an autocrine TGF-β/TGF-βR1 signaling axis activated in 3D spheroids, we next questioned whether glycolytic inhibition affected TGF-β signaling and cell chemotaxis. Cells were treated with increasing concentrations of 2DG in the presence or absence of TGF-β. Intracellular ATP depletion was confirmed (Figure S1), and Western blot analysis showed that TGF-β strongly induced phosphorylation of Smad2 and Smad3, whereas 2DG dose-dependently reduced P-Smad2 and P-Smad3 levels without altering total Smad protein abundance (Figure 4A). Functional assays revealed that TGF-β significantly enhanced cell migration over 2 hours, and that this effect was dose-dependently suppressed by 2DG, with near-complete inhibition at concentrations ≥ 300 μM (Figure 4B). These findings indicate that glycolytic activity is required for optimal TGF-β signaling and migration, linking metabolic regulation to invasive behavior. Because 2DG can induce endoplasmic reticulum (ER) stress and impair N-linked glycosylation in addition to inhibiting glycolysis, we interpret ATP depletion as evidence of energetic stress rather than a specific blockade of a single metabolic pathway; ATP measurements in 2D monolayers were used to establish effective dosing and timing for subsequent 3D spheroid experiments, and follow-up assays to resolve the contributing mechanisms are planned.

### Glycolytic inhibition blocks TGF-β-induced EMT and activates ER stress

**Figure 5:**
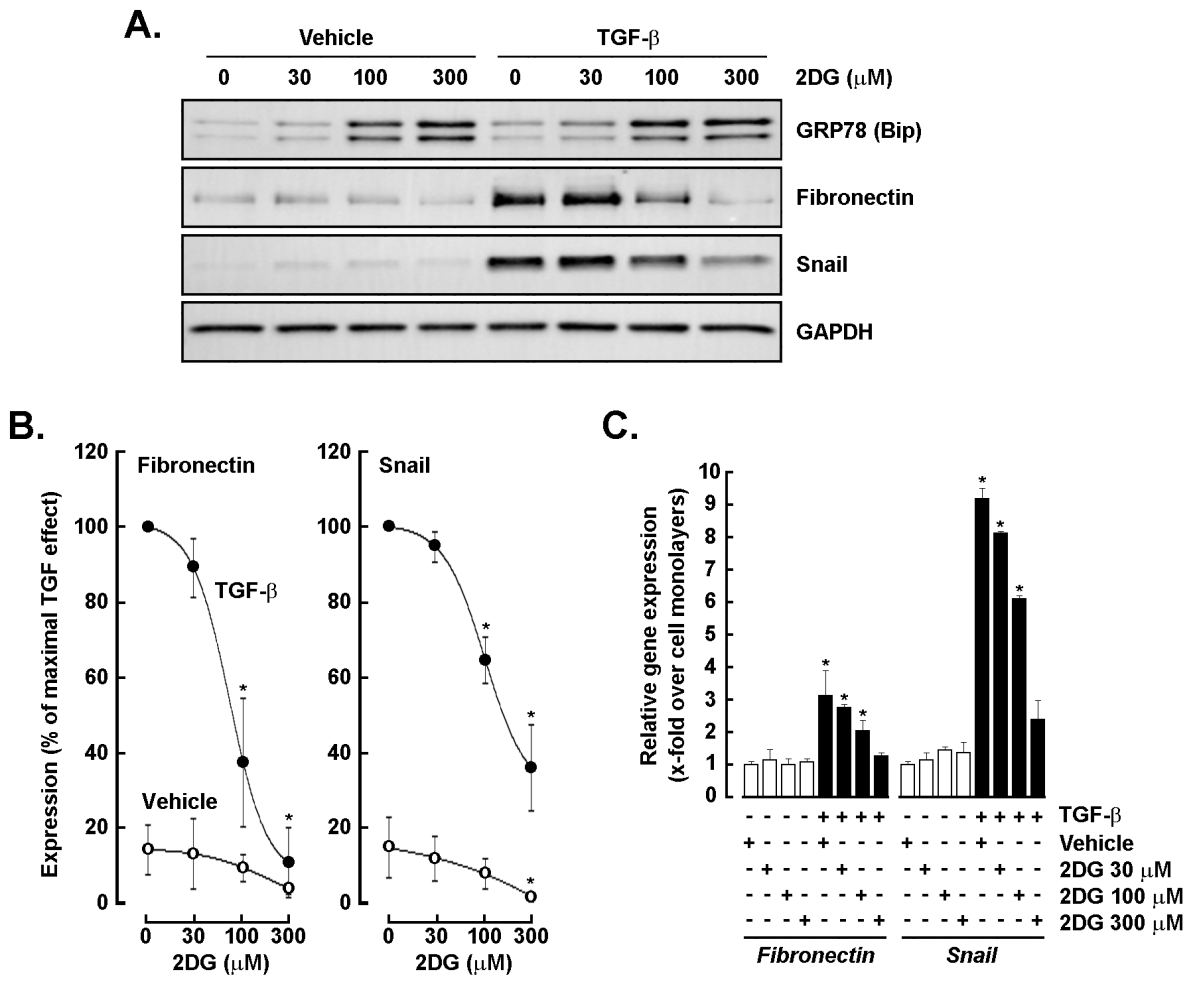
**Glycolytic inhibition suppresses TGF-β-induced EMT marker expression and induces ER stress.** (**A**) Western blot analysis of GRP78 (BiP), fibronectin, and Snail expression levels in cells treated with vehicle or TGF-β (30 ng/mL) in the presence of increasing concentrations of 2-deoxy-*D*-glucose (2DG; 0–300 μM). GAPDH served as a loading control. (**B**) Quantitative analysis of fibronectin and Snail protein levels expressed as a percentage of TGF-β-stimulated control. Densitometry analysis was performed in three independent experiments. (**C**) RT-qPCR analysis of *fibronectin* and *Snail* mRNA expression under the same indicated conditions. All RT-qPCR data are expressed as fold change relative to control and performed in triplicate from two independent experiments. *P* < 0.05 (*) values are considered significant. TGF-β: transforming growth factor-β; EMT: epithelial-mesenchymal transition; ER: endoplasmic reticulum.

To determine whether glycolytic inhibition affects TGF-β-induced EMT, cells were treated with increasing concentrations of 2DG in the presence or absence of TGF-β. Western blot analysis showed that TGF-β markedly increased fibronectin and Snail protein expression, confirming EMT activation, whereas 2DG dose-dependently suppressed both markers (Figure 5A, middle panels). In contrast, GRP78 (BiP), an ER stress marker, was strongly upregulated at higher 2DG concentrations, indicating metabolic stress (Figure 5A, upper panel). Quantitative analysis revealed near-complete inhibition of fibronectin and Snail protein levels at ≥ 300 μM 2DG (Figure 5B), and RT-qPCR confirmed a similar dose-dependent reduction in their mRNA transcripts (Figure 5C). These findings demonstrate that glycolytic activity is required for TGF-β-driven EMT, and its inhibition antagonizes EMT signaling while inducing stress responses.

### Comprehensive analysis of gene expression modulation and functional interactions in response to TGF-β treatment and in the genesis of 3D spheroids

**Figure 6:**
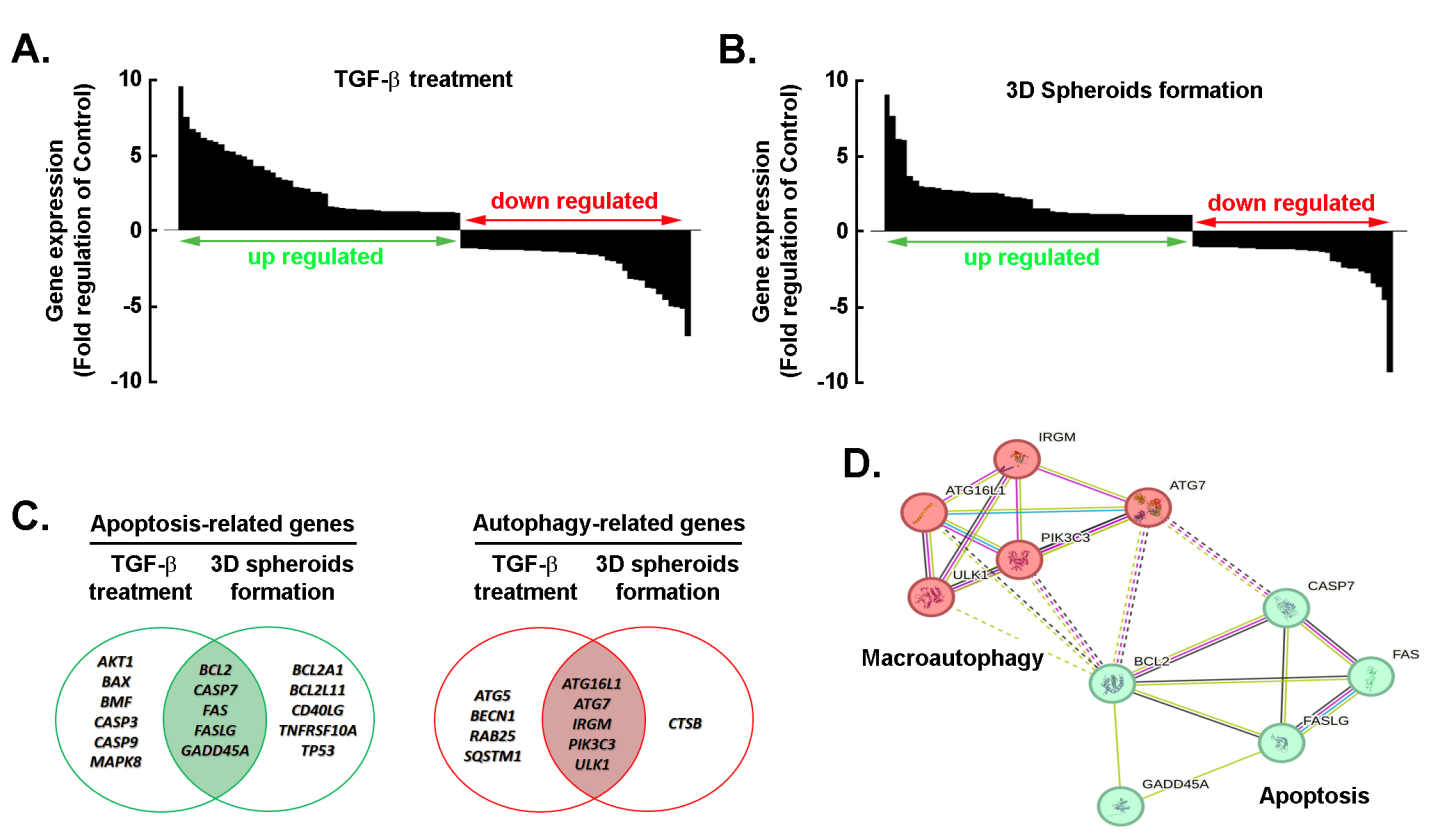
**Comprehensive analysis of gene expression changes and functional interactions under TGF**-β **treatment and 3D spheroid formation.** Waterfall plots showing differential cell death-related gene expression profiles after TGF-β treatment (**A**) and 3D spheroid formation (**B**) compared to the 2D monolayer control. Genes were ranked by fold-change (log_2_ scale), with upregulated genes (log_2_ FC ≥ 2, *P* < 0.05) highlighted in green and downregulated genes (log_2_ FC ≤ –2, *P* < 0.05) in red. (**C**) Venn diagrams illustrating the overlap of apoptosis-related genes (left) and autophagy-related genes (right) between TGF-β treatment and 3D spheroid conditions. (**D**) Protein–protein interaction network generated using the STRING database (confidence score ≥ 0.7) for selected genes involved in macroautophagy and apoptosis. Nodes represent proteins, edges indicate functional associations, and color intensity reflects interaction confidence. Clustering reveals strong connectivity between autophagy regulators and apoptosis mediators, suggesting pathway crosstalk. TGF-β: transforming growth factor-β.

We next explored whether a common TGF-β-mediated molecular signature linked cell death pathways between 2D TGF-β-treated cell monolayers and 3D spheroids. Total RNA was isolated from each condition, and gene arrays were performed as described in the Methods section. Overall, the waterfall plots showed differential cell death-related gene expression profiles after TGF-β treatment (Figure 6A) and 3D spheroid formation (Figure 6B) compared to the 2D monolayer control, with upregulated genes highlighted in green and downregulated genes in red. Accordingly, Venn diagrams further illustrate the overlap of apoptosis-related genes (Figure 6C, left) and autophagy-related genes (Figure 6C, right) between TGF-β treatment and 3D spheroid conditions. Among the upregulated apoptotic genes, *BCL2*, *CASP7*, *FAS*, *FASLG*, and *GADD45A* were triggered by both TGF-β and in 3D spheroids. Similarly, commonly induced autophagic genes included *ATG7*, *ATG16L1*, *IRGM*, *ULK1*, and *PIK3C3*. Protein–protein interaction network, generated using the STRING database (confidence score ≥ 0.7) for the commonly selected genes involved in macroautophagy (Figure 6D, red) and apoptosis (Figure 6D, green), showed functional associations and clustering that revealed strong connectivity between autophagy regulators and apoptosis mediators, suggesting pathway crosstalk.

### Impact of metabolic and signaling modulation on apoptosis- and autophagy-related gene expression in 2D and 3D cultures

**Figure 7:**
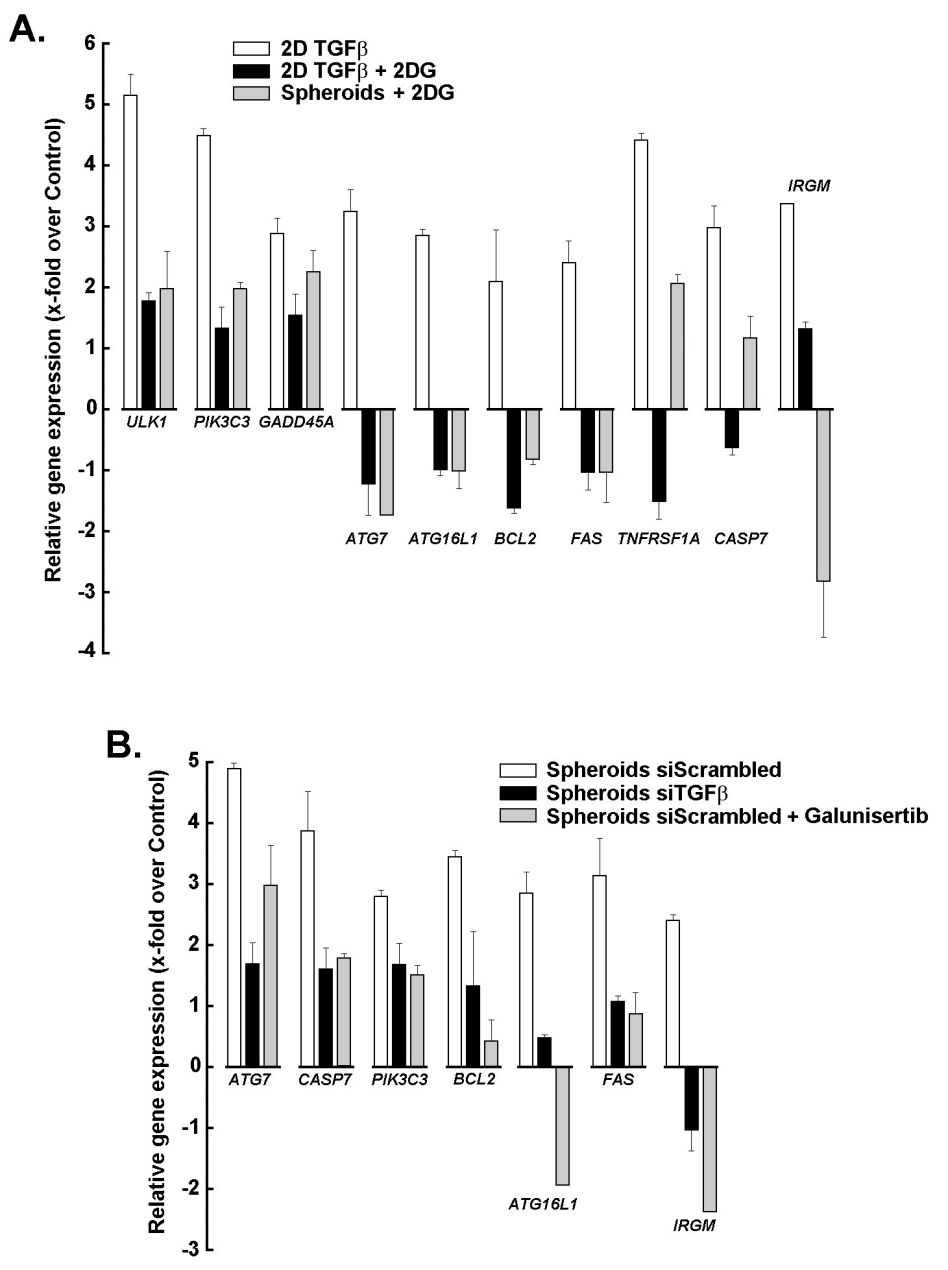
**Impact of metabolic and signaling modulation on apoptosis- and autophagy-related gene expression in 2D and 3D cultures.** (**A**) Relative expression of apoptosis- and autophagy-related genes in 2D cultures treated with TGF-β (white bars), 2D cultures treated with TGF-β + 2DG (black bars), and 3D spheroids treated with 2DG (grey bars), compared to untreated control. (**B**) Relative gene expression changes in 3D spheroids under different conditions: scrambled siRNA (white bars), siTGFβ (black bars), and galunisertib treatment (grey bars). Bars represent mean fold-change relative to control, whereas negative values indicate downregulation. Experiments are the means of two experiments, each performed in triplicate. TGF-β: transforming growth factor-β; 2DG: 2-deoxy-*D*-glucose.

The relative expression of apoptosis- and autophagy-related genes was next assessed in 2D cultures treated with TGF-β (Figure 7A, white bars), 2D cultures treated with TGF-β + 2DG (Figure 7A, black bars), and 3D spheroids treated with 2DG (Figure 7A, grey bars), and compared to the untreated 2D monolayer control. All were found to be sensitive to 2DG-mediated depletion of ATP, and their expression was inhibited. Particularly, the expression of the autophagic marker *ATG7* and the key apoptosis-related genes *BCL2* and *FAS* were found to be differentially regulated. Gene expression changes were similarly assessed in 3D spheroids from scrambled siRNA-transfected cells (Figure 7B, white bars), siTGFβ-transfected cells (Figure 7B, black bars), and upon galunisertib treatment (Figure 7B, grey bars). We found that altering the TGF-β/TGF-βR1 signaling axis significantly prevented these increases, supporting the interplay between TGF-β signaling, glycolytic inhibition, and TGF-βR1 blockade in regulating cell death and survival pathways in 3D spheroids.

## Discussion

The acquisition of apoptotic and autophagic molecular signatures can paradoxically contribute to chemoresistance, despite these pathways being traditionally associated with cell death [31, 32]. Here, we provide novel evidence that TGF-β signaling could potentially play a dual role in promoting the acquisition of an invasive phenotype and in regulating cell death pathways, thereby potentially contributing to drug resistance mechanisms [33]. Indeed, TGF‑β treatment of U87 cells induced robust Smad2 phosphorylation and upregulation of Snail and fibronectin, consistent with activation of a mesenchymal/ECM‑remodeling program. Because U87 is a GBM (neuroepithelial) cell line that typically exhibits low or absent E‑cadherin and constitutive expression of certain mesenchymal markers, we describe these changes as a “TGF-β-induced EMT‑like” or “mesenchymal phenotypic shift” rather than a canonical EMT, and recommend cautious use of EMT terminology unless corroborated by lineage‑appropriate marker dynamics or functional assays (migration, invasion, ECM deposition). More importantly, we demonstrate that such a molecular signature is, in part, recapitulated in 3D GBM spheroid cultures and requires TGF-β signaling [8]. Autophagy in particular, although capable of inducing cell death, often serves as a cytoprotective mechanism under stress conditions such as chemotherapy to facilitate the removal of damaged organelles and proteins, to reduce oxidative stress, and to maintain energy homeostasis [34, 35]. Here, we further establish that these events are mimicked particularly within the in vitro hypoxic nutrient-deprived environment as that generated within 3D spheroids.

Interestingly, we further evidence here that GBM cells may concomitantly trigger an autocrine TGF-β-regulated pro-apoptotic molecular signature, resulting in a primed state that can be regulated upon hypoxia originating from within the 3D spheroids. In GBM spheroids, the 3D architecture is thought to better mimic the tumor microenvironment, including hypoxia and cell-cell interactions, than 2D monolayers, which activate survival pathways such as PI3K/AKT and NF-κB, further enhancing apoptosis-/autophagy-mediated chemoresistance [36, 37]. Accordingly, genome-wide CRISPR/Cas9 screens have identified key regulators of autophagy and apoptosis that could contribute to drug resistance [38]. Selective autophagy was further found to target and regulate distinct signaling pathways that can sensitize cells to chemotherapy [39–41]. Collectively, the interplay between TGF-β signaling, autophagy, and apoptosis appears to be well recapitulated within the in vitro 3D GBM spheroids and supports, in part, the complex adaptive response that enables GBM to withstand chemotherapeutic stress. Our current in vitro validation of such a 3D culture model will help to better understand these mechanisms and to develop novel strategies that would overcome resistance and improve therapeutic outcomes.

Here, we provide evidence that intracellular ATP levels play a pivotal role in the formation and maintenance of 3D spheroids, as these structures appear to require substantial energy to sustain cell-cell adhesion, cytoskeletal remodeling, and signaling networks that drive compaction and viability [22]. Not only is ATP known to fuel actin polymerization and integrin-mediated adhesion, two key processes essential for spheroid architecture and mechanical integrity, but it also supports ion transport for pH and osmotic balance, which becomes critical as spheroids develop hypoxic and acidic cores [42]. In addition, our results show that autophagy, an ATP-dependent process vital for recycling cellular components and maintaining metabolic homeostasis, is increased within the hypoxic microenvironment of 3D GBM spheroids. Depletion of ATP compromised such an adaptive mechanism, leading to spheroid disintegration and increased susceptibility to apoptotic cell death. Thus, ATP availability is a critical determinant of spheroid integrity and of the physiological relevance of 3D tumor models, which again more accurately replicate in vivo tumor heterogeneity and drug resistance compared to 2D cultures [43, 44].

ATP depletion, through the concomitant inhibition of glycolysis and accumulation of G6P following 2DG treatment, appears to impact TGF-β-mediated signaling and stress adaptation. Indeed, 2DG, a glycolytic inhibitor, is phosphorylated by hexokinase but cannot proceed through glycolysis [45], leading to intracellular 2DG6P buildup and feedback inhibition of hexokinase activity [46, 47]. Here, we show that such metabolic blockade impairs phosphorylation-dependent signaling events essential for TGF-β/Smad and PI3K/AKT pathway activation and downstream transcriptional responses [48]. Furthermore, as elevated G6P can disrupt N-linked glycosylation in the ER, causing protein misfolding and ER stress [49], one could hypothesize that this also compromises TGF-β receptor maturation and Smad trafficking. Collectively, these defects attenuate TGF-β-driven transcriptional programs that normally promote autophagy in part through genes identified here and including *PIK3C3*, *ATG7*, *ATG16L1*, and the stress-tolerance gene *GADD45A*, leaving cells unable to recycle nutrients or repair damage under hypoxic conditions. PIK3C3, a class III PI3K, initiates autophagosome formation, while ATG7 and ATG16L1 mediate elongation and maturation of autophagic vesicles. Finally, the upregulation of apoptosis-related genes in spheroids is spatially restricted, with anti-apoptotic *BCL2* favoring spheroid integrity while *FAS* and *CASP7* may contribute to necrotic core formation.

Physiologically, this dual impact, metabolic stress combined with impaired TGF-β signaling, undermines GBM’s adaptive capacity, particularly in 3D spheroid models where hypoxia and nutrient gradients intensify reliance on these pathways. It becomes tempting to further suggest that G6P accumulation is not merely a simple metabolic consequence of 2DG treatment but rather a potential critical mediator of signaling disruption, reinforcing the therapeutic impact of metabolic inhibitors in combination with TGF-β antagonists. Collectively, these observations underscore the importance of 3D models in preclinical drug testing. Unlike 2D cultures, spheroids replicate hypoxic and heterogeneous conditions that drive TGF-β-mediated metabolic rewiring and stemness acquisition, both of which are critical determinants of chemoresistance. Targeting these adaptive pathways, particularly autophagy and TGF-β/HIF-1α signaling, may offer novel therapeutic strategies for GBM. While 3D tumor models used here recapitulate critical aspects of the glioma microenvironment and provide mechanistic and translationally relevant data, we acknowledge that intracranial implantation studies are the definitive test of in vivo relevance. To minimize animal use and maximize experimental rigor, we decided to first establish reproducible effects in 3D systems. Focused intracranial xenograft experiments to validate these findings in vivo will be required.

In conclusion, we show that 2DG-mediated inhibition of glycolysis imposes severe energetic stress and suppresses TGF-β-mediated transcriptional regulation, which may prevent activation of compensatory survival pathways. Physiologically, this dual blockade deprives GBM cells of invasiveness, autophagic recycling, stress tolerance, and apoptotic regulation, forcing them into metabolic collapse and eventually sensitizing them to therapeutic intervention. It must be acknowledged that these transcriptomic data are hypothesis-generating; confirmation of apoptosis and autophagy will require protein-level and flux assays. These transcriptomic data are hypothesis‑generating; targeted biochemical and functional assays (e.g., LC3‑II flux, p62 degradation, cleaved caspase‑3, annexin V/TUNEL) are required to confirm pathway activation and are planned for follow‑up validation. We therefore interpret the observed pro-apoptotic and autophagy-related transcriptional signatures as indicators of pathway engagement that warrant targeted functional validation, which we plan to perform in U87 as well as in additional GBM models, including patient-derived stem-like cultures.

## Abbreviations

2DG: 2-deoxy-*D*-glucose
EMT: epithelial-mesenchymal transition
ER: endoplasmic reticulum
G6P: glucose-6-phosphate
GBM: glioblastoma
P-Smad2: phospho-Smad2
RT-qPCR: real-time quantitative PCR
SDS: sodium dodecylsulfate
siRNA: small interfering RNA
TGF-β: transforming growth factor-β

## Supplementary materials

The supplementary figure for this article is available at: https://www.explorationpub.com/uploads/Article/file/1002364_sup_1.pdf and the supplementary table for this article is available at: https://www.explorationpub.com/uploads/Article/file/1002364_sup_2.xlsx.

## Declarations

### Availability of data and materials

The datasets used and/or analysed during the current study are available from the corresponding author on reasonable request.

### Copyright

© The Author(s) 2026.
