# Supplementary material for: Disrupting the TGF-β-regulated epithelial-mesenchymal transition, apoptotic and autophagic phenotypes of 3D glioblastoma spheroids via glycolytic inhibition
Source: Explor Target Antitumor Ther. 2026 Mar 30;7:1002364. doi: 10.37349/etat.2026.1002364 (PMC13087734; doi:10.37349/etat.2026.1002364)
Supplement: Supplementary file 1 [file 1002364_sup_1.pdf]

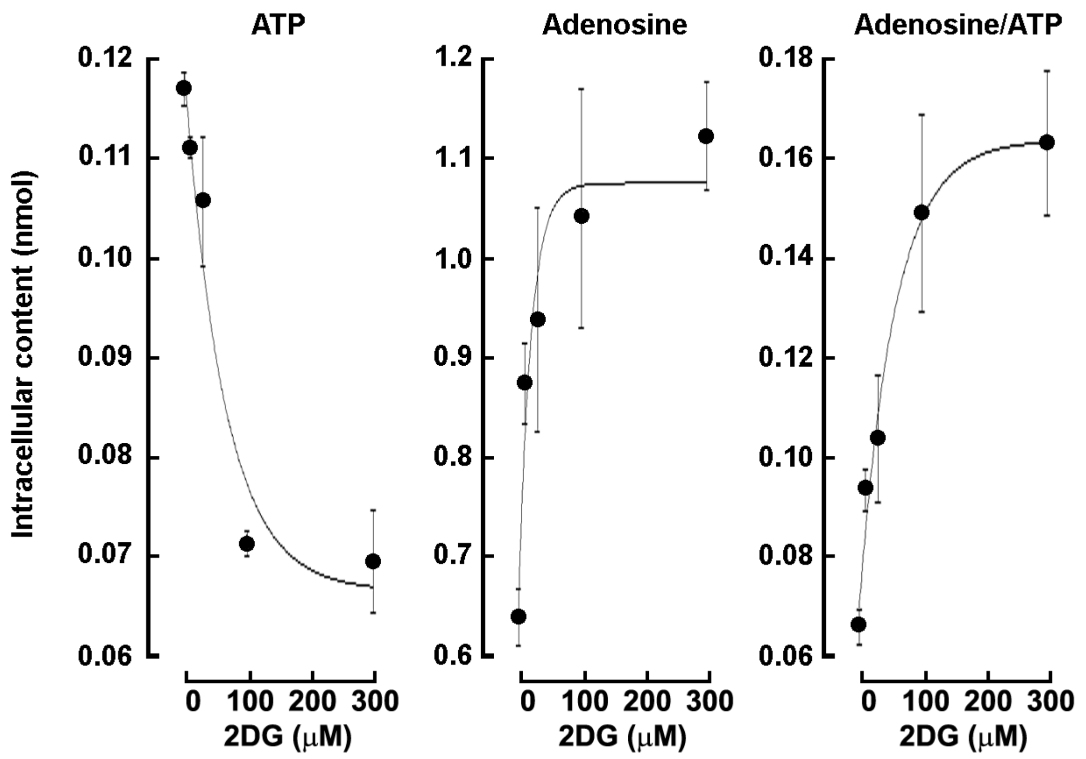

Figure S1. Glycolytic inhibition by 2-deoxy-*D*-glucose depletes intracellular ATP content in 2D monolayers. Quantitative intracellular content of ATP demonstrates its dose-dependent depletion by 2DG (10–300 μM, left panel). The middle panel shows intracellular Adenosine content increasing upon 2DG treatment, reaching a plateau around 100 μM. Finally, the right panel represents the Adenosine/ATP ratio with the indicated increasing concentrations of 2DG, demonstrating depletion of ATP and its replacement by Adenosine. This validates the impact of 2DG metabolic inhibitor capacity.
